# Effects of Dietary Protein Levels on Fecal Amino Acids Excretion and Apparent Digestibility, and Fecal and Ileal Microbial Amino Acids Composition in Weaned Piglets

**DOI:** 10.3389/fnut.2021.738707

**Published:** 2021-12-16

**Authors:** Zhenguo Yang, Huan Deng, Tianle He, Zhihong Sun, Ziema Bumbie Gifty, Ping Hu, Zebing Rao, Zhiru Tang

**Affiliations:** ^1^Laboratory for Bio-Feed and Molecular Nutrition, College of Animal Science and Technology, Southwest University, Chongqing, China; ^2^Chongqing Key Laboratory of Herbivore Science, Chongqing, China

**Keywords:** low protein diet, intestinal microorganism, amino acids, apparent digestibility, weaned piglets

## Abstract

**Background and Aims:** The purpose of this study was to determine the effects of low protein diets with the same Lys, Met + Cys, Thr, and Trp levels as in high protein diets on the fecal amino acid excretion and apparent digestibility, and ileal and fecal microbial amino acids composition in weaned piglets.

**Methods:** Fifty-four 21-day-old Duroc × Landrace × Yorkshire weaned piglets were randomly divided into three groups and fed with corn-soybean meal basal diets, in which the crude protein (CP) content was 20% (H-CP), 17% (M-CP), and 14% (L-CP), respectively. The experiment included a 7-day adaptation period and a 45-day trial period. Six piglets in each group were randomly slaughtered on days 10, 25, and 45 of the trial period, and the intestinal contents, intestinal mucosa, and feces were collected.

**Results:** The results showed that the interaction between feeding time and dietary CP levels was reflected in the apparent digestibility of dietary CP and amino acid (AA) (*p* < 0.01). With the increase of age, the apparent digestibility of CP and AA were increased (*p* < 0.01). With the increase of CP levels, the excretion of nitrogen (N) was decreased (*p* < 0.01), whereas the flow of microbial AA in the ileum and feces were increased (*p* < 0.01). The interaction between feeding time and dietary CP levels was also reflected in the composition of AA in the ileum and stool of piglets (*p* < 0.01). The proportion of His, Lyr, Met, Cys, and Ser was lower than the average, whereas the proportion of Phe, Leu, Pro, Ala, Glu, and Asp was higher than the average. With the increase of age, the AA content of microorganisms increased (*p* < 0.01).

**Conclusion:** All in all, this work revealed the changes of N, CP, and AA excretion and digestibility of feces and microorganisms of piglets under the combined action of different dietary protein levels and different feeding times, and also the changes of AA composition of intestinal microorganisms and AA composition of microorganisms.

## Introduction

The shortage of protein feed resources and the environmental pollution caused by N emissions were two difficult problems to be solved urgently in the piglets' industry nowadays (1). A low protein diet with an AA balance could effectively alleviate these two problems and be gradually applied to different growth stages of piglets. From the economic and environmental point of view, reducing dietary CP and supplementing crystalline AA were effective strategies for the piglets' industry to reduce costs and pollution (2, 3). It was reported that for every 1% reduction of CP in the diet, the total N excretion was reduced by about 8% (4). According to the above theory and the NRC recommendation, reducing the dietary CP level by 2–4%, according to the NRC recommendation, and adding appropriate synthetic AA could not only meet the protein demand of animals but also effectively reduce N emission (5). The researchers lowered feed CP levels according to theoretical values and added crystalline Lys, Try, Thr, and Met to the feed and found that such a combination did not cause a decline in the growth performance of the piglets (6). Dai et al. (7) also pointed that this AA could make up for the lack of feed protein. Therefore, to maximize the saving of protein resources and alleviate pollution, the critical point of dietary CP levels should be determined and the best AA balance model should be designed.

Ignoring the effect of intestinal microorganisms on protein digestion and metabolism, most traditional nutrition studies focused on the intake of protein, AA patterns, and protein utilization of piglets themselves (8). The microorganisms in piglet intestines were mainly anaerobes and facultative anaerobes, of which Bacteroides account for more than 90%, which played an important role in maintaining body health and improving immunity, absorption, and metabolism of nutrients (9). Dietary proteins and AA which could not be digested in the small intestine (together with nitrogenous substances such as digestive enzymes and mucus secreted by the small intestine) were used by microorganisms to synthesize bacterial proteins after entering the intestine or degrade metabolites such as ammonia, hydrogen sulfide, biogenic amines, phenols, and indole compounds (10). The decrease of dietary protein level in growing piglets increased the relative abundance of intestinal microflora (11). The intestinal microorganisms of porcine metabolized some essential AA (such as Lys and Thr) and some conditionally essential AA, and the metabolism of some specific AA required the interaction of some specific microorganisms (7). Therefore, intestinal bacteria colonized their hosts in the process of N metabolism.

However, there was no in-depth research on the metabolism of AA and proteins by intestinal microorganisms. There were few reports about the effect of CP level on the AA composition of intestinal bacteria. Therefore, the experiment was conducted to study the effects of dietary protein levels on the apparent digestibility of CP and AA and the AA composition of intestinal microorganisms in piglets. Thus, this study provided a scientific theoretical basis for further understanding the digestion and absorption of protein in the intestinal tract of piglets, reducing N emissions, and changing the supply mode of N nutrients by gastrointestinal microorganisms. The objectives of the present work were to investigate whether the same levels of Lys, Met + Cys, Thr, and Trp in the L-CP diet and the H-CP diet affected fecal amino acid excretion, apparent digestibility, and microbial amino acid composition in ileum and feces of weaned piglets.

## Materials and Methods

### Animals, Experimental Design, and Diets

Fifty four Duroc × Landrace × Yorkshire barrows (initial BW = 5.55 ± 0.49 kg, 21-day-old) were obtained from a local commercial swine farm. All piglets were raised separately in 24 ± 1°C steel metabolic crates. Fifty-four piglets were randomly divided into three groups, with 18 piglets in each group and each piglet was a repeat. The experiment lasted for 45 days according to three dietary protein levels (14, 17, and 20%). The diets for piglets were prepared according to various nutrients recommended by NRC (12). They were allowed to eat and drink freely in a mechanically ventilated temperature-controlled room at 24 ± 1°C. The formula of the diet is shown in Table 1. All experimental procedures were approved by the License of Experimental Animals (SYXK 2014-0002) of the Animal Experimentation Ethics Committee of Southwest University, Chongqing, China.

**Table 1:** Ingredient composition of experimental diets in weaned piglets (g/kg, DM basis).

**Ingredients**	**14%CP**	**17%CP**	**20%CP**
Corn	71.80	66.50	63.70
Soybean meal	13.40	18.80	19.80
Whey powder	4.40	4.30	4.30
Fish meal	1.50	4.00	9.00
Soybean oil	4.10	2.60	0.80
L-Lys·HCl	0.88	0.62	0.38
DL-Met	0.27	0.19	0.10
L-Thr	0.33	0.21	0.09
L-Trp	0.08	0.04	0.01
Monocalcium phosphate	1.15	0.74	0.00
Limestone	0.79	0.70	0.52
Salt	0.30	0.30	0.30
Premix	1.00	1.00	1.00
Total	100.00	100.00	100.00
**Nutritional level (based on chemical analysis)**			
DM (MJ/kg)	14.60	14.60	14.60
CP	14.14	17.32	20.27
Lys	1.26	1.25	1.26
Met + Cys	0.63	0.65	0.62
Thr	0.76	0.75	0.76
Trp	0.20	0.20	0.20
Arg	0.71	0.93	1.09
His	0.30	0.37	0.44
Ileu	0.46	0.60	0.71
Leu	1.11	1.32	1.52
Phe	0.56	0.70	0.81
Val	0.54	0.64	0.72
Ca, %	0.70	0.71	0.69
Available *P*, %	0.53	0.55	0.57
EAA	6.29	7.18	7.91
NEAA	6.84	8.40	9.74
EAA/NEAA	0.90	0.85	0.80

*In addition to digestive energy data, the remaining nutrients are measured values*.

### Measurements and Sampling

**Figure 1:**
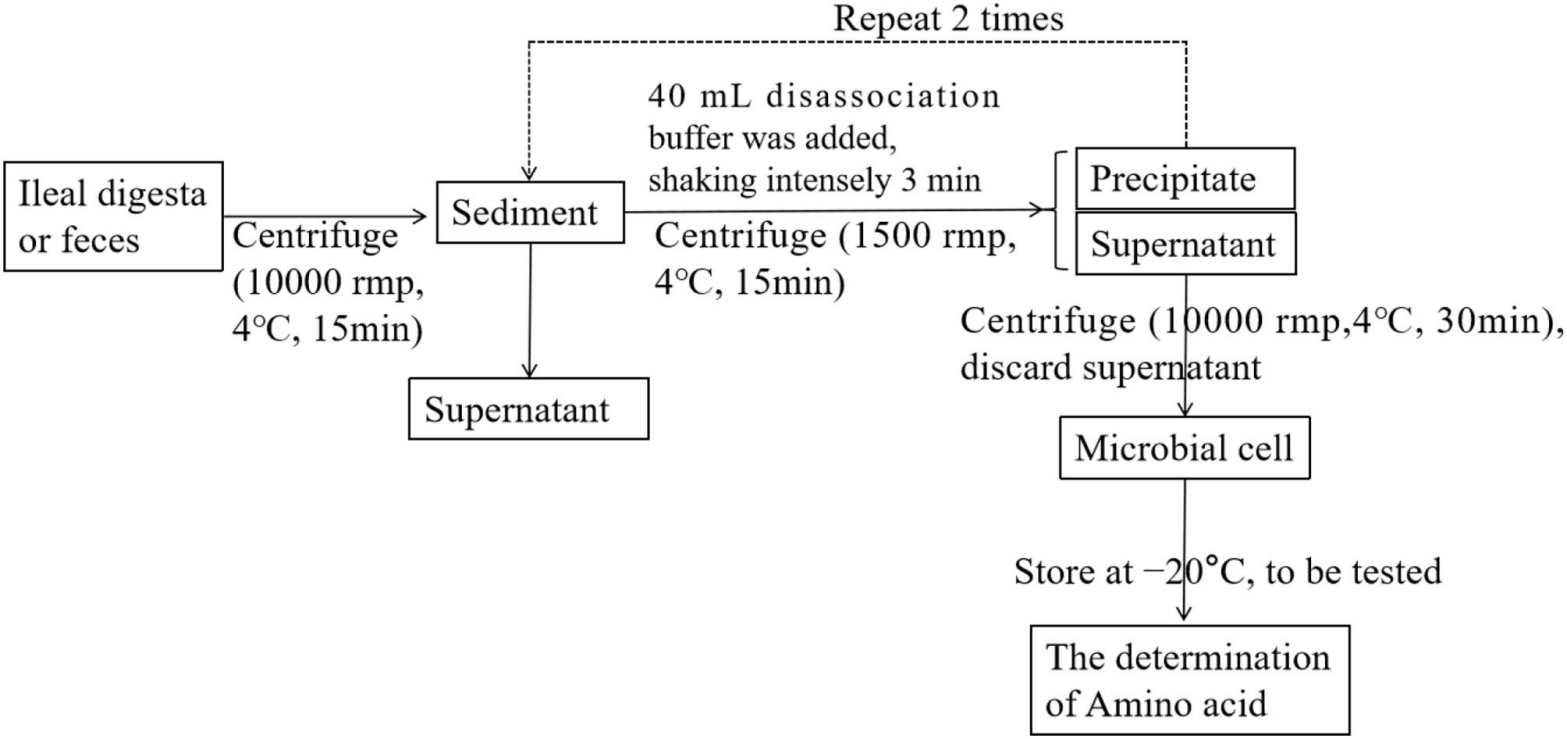
Schematic diagram of the processing of the digesta samples.

On days 10, 25, and 45 of the experiment, six piglets were randomly selected from each group to collect feces, and then intravenous anesthesia with pentobarbital sodium (50 mg/kg BW) was used to collect blood. In addition, the digestive juice at the end of the ileum was collected with sterilized plastic bags. The samples were stored at 4°C for microbial separation and chemical analysis. A summary of the centrifugation protocol is given in Figure 1. The samples for chemical analysis were stored at −20°C (6).

### Chemical Analyses

The feces were dried to a constant mass in a forced bellow at 95°C, and other digestive juice samples were fractionated by 250 RCF for 15 min at 4°C differential centrifugation. The determination of total N and AA composition follows the method proposed by predecessors (9, 10).

### Data Treatment and Statistical Analysis

The endogenous indicator acid insoluble ash (AIA) was determined to calculate the apparent digestibility of dry matter, CP, and total AA. The formula is as follows: 1-bc/ad, where *a* denotes the content of DM, CP, or AA in diets (%); *b* denotes the content of dry matter (DM), CP, or AA in feces (%); *c* denotes the content of AIA in diet (%); and *d* denotes the content of AIA in feces (%). AA compositions were the content of single AA in TAA. The basic statistics of the data were carried out by Microsoft Excel 2020, the routine indexes were analyzed by SAS two-way ANOVA and significance test, and the measured data were analyzed by single factor ANOVA and significance test by SAS statistical software. The results were expressed by mean ± SEM, *p* < 0.05. The difference was significant.

## Results

### Fecal N Excretion and Digestibility

As shown in Table 2, the excretion and digestibility of DM, CP, TN, total protein N (TPN), and total non-protein N (TNN) of piglets were increased with the increase of dietary protein levels on days 10 and 25 (*p* < 0.01). The excretion and digestibility of DM, CP, TN, TPN, and TNN of L-CP and H-CP were higher than those in the M-CP group on day 45 (*p* < 0.01). The excretion and digestibility of DM, CP, TN, TPN, and TNN of L-CP and H-CP were higher than those in the M-CP group on day 45 (*p* < 0.01). The excretion of microbial total N (MTN), microbial protein N (MPN), and microbial non-protein N (MNN) of piglets in the H-CP group were higher than those in the L-CP group and M-CP group on day 10 (*p* < 0.01). The excretion of MTN, MPN, and MNN in the M-CP group was higher than those in the L-CP group and H-CP group on days 25 and 45 (*p* < 0.01). The excretion of MTN, MTN, and MNN at day 25 was lower than those on days 10 and 45 (*p* < 0.01). The interaction effects between feeding time and dietary protein level (Days × CP) were as follows: CP (*p* < 0.01); total N (TN) (*p* < 0.01); TPN (*p* < 0.01); total non-protein N (TNN) (*p* < 0.01); MTN (*p* < 0.01); MPN (*p* < 0.01); and MNN (*p* < 0.01).

**Table 2:** Effects of dietary different protein levels on N excretion in feces and its apparent digestibility of piglets.

**Time (d)**	**Items**	**DM-D (%)**	**CP-flow (mg/g DM)**	**CP-D (%)**	**TN (mg/g DM)**	**TN-D (%)**	**TPN (mg/g DM)**	**TPN-D (%)**	**TNN (mg/g DM)**	**TNN-D (%)**	**MTN (mg/g DM)**	**MPN (mg/g DM)**	**MNN (mg/g DM)**
10 d	L-CP	85.0	261	69.6	41.8	69.6	36.7	76.5	5.1	58.5	0.67	0.59	0.09
	M-CP	83.7	282	70.9	45.1	70.9	40.1	74.3	5.0	54.7	1.34	1.23	0.11
	H-CP	89.6	302	82.6	48.3	82.6	42.8	84.4	5.5	75.4	2.12	1.86	0.26
25 d	L-CP	88.9	236	79.7	37.8	79.7	33.4	84.1	4.4	73.6	0.44	0.39	0.05
	M-CP	88.8	271	80.8	43.4	80.8	38.0	83.5	5.4	66.0	0.94	0.84	0.11
	H-CP	89.1	279	83.3	44.6	83.3	39.1	85.5	5.5	73.8	0.61	0.51	0.11
45 d	L-CP	91.2	228	84.2	36.6	84.2	31.0	88.3	5.6	77.2	1.35	1.17	0.17
	M-CP	88.6	208	84.1	33.3	84.1	28.1	88.4	5.2	75.6	1.86	1.67	0.19
	H-CP	90.0	224	87.8	35.9	87.8	30.7	90.5	5.2	81.5	1.63	1.44	0.19
SEM	Days	0.38	3.0	0.59	0.48	0.59	0.47	0.49	0.07	0.63	0.018	0.015	0.005
	CP	0.38	3.0	0.59	0.48	0.59	0.47	0.49	0.07	0.63	0.018	0.015	0.005
	Days × CP	0.66	5.2	1.03	0.8	1.03	0.8	0.85	0.12	1.09	0.028	0.025	0.008
*P*-Value	Days	<0.01	<0.01	<0.01	<0.01	<0.01	<0.01	<0.01	0.08	<0.01	<0.01	<0.01	<0.01
	CP	<0.01	<0.01	<0.01	<0.01	<0.01	<0.01	<0.01	<0.01	<0.01	<0.01	<0.01	<0.01
	Days × CP	<0.01	<0.01	<0.01	<0.01	<0.01	<0.01	<0.01	<0.01	<0.01	<0.01	<0.01	<0.01

*L-CP, piglets fed a maize-soybean meal diet containing 14% CP; M-CP, piglets fed a maize-soybean meal diet containing 17% CP; H-CP piglets fed a maize-soybean meal diet containing 20% CP; SEM, standard error means; DM, dry matter; CP, crude protein; TN, total nitrogen, TPN, total protein nitrogen; TNN, total non-protein nitrogen; MTN, microbial total nitrogen; MPN, microbial protein nitrogen; MNN, microbial non-protein nitrogen. The same is in the following tables*.

### Fecal Total AA Excretion and Apparent Digestibility

As shown in Table 3, the excretion of total AA (TAA), essential AA (EAA), and non-essential AA (NEAA) decreased with the increase of feeding days of piglets (*p* < 0.01), and the digestibility of TAA, EAA, and NEAA increased with the prolongation of feeding time (*p* < 0.01). The excretion of TAA, EAA, NEAA, TAA (–M), NEAA (–M), and EAA (–M) increased with the increase of dietary protein level on days 10 and 20 (*p* < 0.01). The digestibility of TAA, EAA, and NEAA in the H-CP group was higher than those in the L-CP group and M-CP group (*p* < 0.01). The digestibility of TAA, EAA, and NEAA in the M-CP group was lower than those in L-CP and H-CP groups at days 10 and 25 (*p* < 0.01). The digestibility of TAA, EAA, and NEAA in the L-CP group was lower than those in M-CP and H-CP groups at day 45 (*p* < 0.01). Interestingly, the change law of the above results is similar after the AA part of the microbes were removed from feces AA. The interaction effects between feeding time and dietary protein level (Days × CP) were as follows: TAA (*p* < 0.01), TAA digestibility (*p* < 0.01), NEAA (*p* < 0.01), NEAA digestibility (*p* < 0.01), EAA digestibility (*p* < 0.0l), TAA (–M) (*p* < 0.01), TAA digestibility (–M) (*p* < 0.01), NEAA (–M) (*p* < 0.01), NEAA digestibility (–M) (*p* < 0.01), EAA (–M) (*p* < 0.01), and EAA digestibility (–M) (*p* < 0.01).

**Table 3:** Effects of dietary protein levels on the TAA excretion (mg/g DM) and apparent digestibility (%) of piglets.

**Time (d)**	**Items**	**TAA (+M)**	**TAA-D (+M, %)**	**NEAA (+M)**	**NEAA-D (+M, %)**	**EAA (+M)**	**EAA-D (+M, %)**	**E/NE (+M)**	**TAA (–M)**	**TAA-D (–M, %)**	**NEAA (–M)**	**NEAA-D (–M, %)**	**EAA (–M)**	**EAA-D (–M)**	**EAA/NEAA (–M)**
10 d	L-CP	226	76.5	111	78.0	115	77.3	1.04	222.2	76.9	109.3	75.98	112.8	77.7	1.03
	M-CP	251	74.3	120	76.5	131	74.4	1.09	243.8	75.1	117.5	74.91	126.6	75.2	1.08
	H-CP	270	84.4	132	84.5	138	83.8	1.05	258.4	85.2	126.9	88.72	131.5	84.6	1.04
25 d	L-CP	207	84.1	102	85.0	105	84.7	1.03	204.5	84.2	101.4	83.52	103.2	84.9	1.02
	M-CP	236	83.5	117	84.3	119	84.1	1.02	231.2	83.9	114.3	83.31	116.3	84.4	1.02
	H-CP	243	85.5	118	85.6	125	84.8	1.06	239.4	85.7	116	86.40	123.5	85.0	1.06
45 d	L-CP	195	88.3	105	87.3	90	88.3	0.86	187.6	88.5	101.9	86.90	85.6	90.0	0.84
	M-CP	179	88.4	87	88.8	92	89.2	1.06	169.0	88.1	82.3	87.83	86.7	88.3	1.05
	H-CP	194	90.5	97	90.5	97	90.4	1.00	184.7	90.1	92.5	90.33	92.2	89.9	1.00
SEM	Days	3	0.49	1.4	0.49	1.70	0.50		3.0	0.48	1.7	0.49	1.8	0.48	
	CP	3	0.49	1.4	0.49	1.70	0.50		3.0	0.48	1.4	0.49	1.7	0.48	
	Days × CP	5.2	0.85	2.4	0.84	3	0.86		5.1	0.83	2.4	0.85	2.9	0.83	
*P*-value	Days	<0.01	<0.01	<0.01	<0.01	<0.01	<0.01	<0.01	<0.01	<0.01	<0.01	<0.01	<0.01	<0.01	<0.01
	CP	<0.01	<0.01	<0.01	<0.01	<0.01	<0.01	<0.01	<0.01	<0.01	<0.01	<0.01	<0.01	<0.01	<0.01
	Days × CP	<0.01	<0.01	<0.01	<0.01	0.06	<0.01	<0.01	<0.01	<0.01	<0.01	<0.01	<0.01	<0.01	<0.01

### Fecal AA Excretion and Apparent Digestibility

As shown in Table 4, the excretion of all amino acids in piglets, except Cys, decreased with the increase of feeding time (*p* < 0.01). The excretion of Asp, Pro, Val, Ile, Leu, Try, Phe, and Lys increased with the increase of dietary protein level (*p* < 0.01). The excretion of Asp, Ser, Glu, Gly, Ala, Cys, Val, Ile, Leu, Lys, Phe, Lys, and Arg increased with the increase of dietary protein level on day 10 (*p* < 0.01). The excretion of Asp, Ser, Gly, Ala, Thr, Cys, Val, Met, Ile, Leu, Try, Phe, Lys, and Arg increased with the increase of dietary protein level on day 25 (*p* < 0.01). The excretion of Asp, Pro, Thr, Val, Ile, Leu, Try, and Phe increased with the increase of dietary protein level (*p* < 0.01) on day 45. The interaction effects between feeding time and dietary protein level (Days × CP) were as follows: Try (*p* < 0.01), Glu (*p* < 0.01), glycine (Gly) (*p* < 0.01), Ala (*p* < 0.01), Pro (*p* < 0.01), Thr (*p* < 0.01), Cys (*p* < 0.01), Met (*p* < 0.01), Lys (*p* < 0.01), His (*p* < 0.01), and Arg (*p* < 0.01) in the feces of piglets.

**Table 4:** Effects of dietary protein levels on the excretion of the amino acid in feces of piglets (mg/g DM).

**Time (d)**	**Items**	**Asp**	**Ser**	**Glu**	**Gly**	**Ala**	**Pro**	**Tyr**	**Thr**	**Cys**	**Val**	**Met**	**Ile**	**Leu**	**Phe**	**Lys**	**His**	**Arg**
10 d	L-CP	22.4	7.1	30.3	13.7	23.1	15.1	8.2	12.4	3.5	15.5	3.2	13.8	24.9	12.2	10.5	4.5	6.1
	M-CP	23.0	8.3	32.7	17.3	24.7	14.3	9.0	11.6	3.8	18.8	4.4	16.4	29.1	14.2	12.0	4.6	7.2
	H-CP	24.9	11.8	35.6	18.6	26.7	14.4	9.5	12.1	5.0	19.1	4.0	17.0	31.4	14.0	12.2	5.0	8.6
25 d	L-CP	19.5	5.8	28.7	14.2	22.1	12.6	5.8	7.8	4.9	15.6	3.3	13.3	23.9	11.1	9.9	3.9	5.0
	M-CP	22.6	7.6	31.6	16.9	24.8	13.5	7.7	10.2	3.7	17.7	3.5	15.5	26.4	11.9	11.4	4.2	6.5
	H-CP	22.5	9.2	30.6	17.7	24.4	13.5	8.3	10.0	8.4	17.8	4.1	15.1	26.7	12.4	11.6	4.1	6.7
45 d	L-CP	13.7	6.3	37.6	16.4	23.4	7.5	5.1	7.5	7.0	11.5	3.7	10.1	16.7	8.6	9.0	5.1	5.6
	M-CP	15.9	5.9	22.8	13.4	19.7	9.4	6.1	7.5	3.6	14.3	2.2	12.1	20.5	9.9	8.1	3.4	4.5
	H-CP	18.0	6.3	37.7	15.4	19.7	12.2	6.7	8.6	3.4	14.5	2.7	12.5	20.9	10.2	9.1	3.3	4.8
SEM	Days	0.4	0.2	0.5	0.3	0.3	0.3	0.2	0.2	0.4	0.2	0.1	0.3	0.5	0.2	0.2	0.1	0.2
	CP	0.4	0.2	0.5	0.3	0.3	0.3	0.2	0.2	0.4	0.2	0.1	0.3	0.5	0.2	0.2	0.1	0.2
	Days × CP	0.70	0.4	0.9	0.5	0.60	0.50	0.4	0.30	0.67	0.4	0.2	0.4	0.83	0.3	0.3	0.2	0.3
*P*-value	Days	<0.01	<0.01	<0.01	<0.01	<0.01	<0.01	<0.01	<0.01	0.02	<0.01	<0.01	<0.01	<0.01	<0.01	<0.01	<0.01	<0.01
	CP	<0.01	<0.01	<0.01	<0.01	0.33	<0.01	<0.01	<0.01	<0.01	<0.01	0.33	<0.01	<0.01	<0.01	<0.01	0.05	<0.01
	Days × CP	0.23	<0.01	<0.01	<0.01	<0.01	<0.01	0.6	<0.01	<0.01	0.46	<0.01	0.57	0.25	0.58	<0.01	<0.01	<0.01

As can be seen from Table 5, the apparent digestibility of Ala, Ile, Leu, Tyr, Phe, and Lys of piglets in the M-CP group was lower than that of the L-CP group and H-CP group (*p* < 0.01). The apparent digestibility of Met was higher than that in the other two groups. The apparent digestibility of Asp, Glu, Gly, Ala, Pro, Thr, Cys, Val, Ile, Leu, Tyr, Phe, Lys, His, and Arg in the H-CP group was higher than those in the L-CP group and M-CP group on day 10 (*p* < 0.01). The apparent digestibility of fecal Cys, Val, and Met in the M-CP group were higher than that in the L-CP group and H-CP group (*p* < 0.01), and the remaining amino acids were lower than those in the other two groups on day 25 (*p* < 0.01). The apparent digestibility of Glu, Gly, Cys, Met, His, and Arg increased with the increase of dietary protein level (*p* < 0.01), and the apparent digestibility of the remaining amino acids in the M-CP group was lower than those in the L-CP group and H-CP group on day 45 (*p* < 0.01). The interaction effects between feeding time and dietary protein level (Days × CP) were as follows: Asp (*p* < 0.01), Glu (*p* < 0.01), Gly (*p* < 0.01), Ala (*p* < 0.01), Pro (*p* < 0.01), Thr (*p* < 0.01), Cys (*p* < 0.01), Val (*p* < 0.01), Met (*p* < 0.01), Ile (*p* < 0.01), Leu (*p* < 0.01), Tyt (*p* < 0.01), Phe (*p* < 0.01), Lys (*p* < 0.01), His (*p* < 0.01), and Arg (*p* < 0.01) in piglet feces. At the level of feeding time, except for Cys and Met, the apparent digestibility of AA in feces increased significantly with the increase of feeding time (*p* < 0.01).

**Table 5:** Effects of dietary protein levels on the AA apparent digestibility in feces of piglets (%).

**Time (d)**	**Items**	**Asp**	**Ser**	**Glu**	**Gly**	**Ala**	**Pro**	**Tyr**	**Thr**	**Cys**	**Val**	**Met**	**Ile**	**Leu**	**Phe**	**Lys**	**His**	**Arg**
10 d	L-CP	71.5	84.1	80.2	72.3	65.3	76.4	69.5	74.3	74.5	70.3	83.7	69.8	76.1	75.9	80.0	87.0	89.3
	M-CP	76.7	79.8	79.0	64.2	60.8	77.1	64.4	74.0	69.9	61.3	92.1	62.7	68.8	70.4	76.4	86.0	87.2
	H-CP	84.9	84.0	87.9	81.5	78.3	88.9	76.5	83.9	80.4	81.1	86.7	79.5	82.1	83.8	85.7	90.8	91.8
25 d	L-CP	81.8	90.3	86.1	78.9	75.6	85.5	83.9	88.2	73.5	75.0	87.5	78.5	83.0	83.9	86.1	91.9	93.5
	M-CP	84.3	87.3	86.0	76.0	73.3	85.3	79.1	84.3	79.6	87.3	95.7	75.9	80.7	83.0	84.6	91.2	92.1
	H-CP	86.0	87.2	89.2	81.5	79.2	89.3	79.1	86.3	66.4	77.9	86.3	81.2	84.2	85.0	85.8	92.6	93.4
45 d	L-CP	91.6	90.4	86.5	80.0	85.4	93.9	87.7	91.2	68.6	87.6	82.6	87.6	89.1	88.9	91.3	89.9	94.4
	M-CP	89.0	90.0	91.2	83.5	84.2	91.4	84.7	89.3	83.2	83.1	92.7	83.6	86.7	87.7	91.2	92.8	95.1
	H-CP	91.0	91.0	92.8	86.6	86.2	91.5	86.1	89.8	87.3	87.3	92.1	87.4	89.8	90.3	91.9	94.6	96.1
SEM	Days	0.59	0.5	0.43	0.65	0.77	0.5	0.85	0.64	1.69	0.68	0.65	0.74	0.56	0.58	0.47	0.44	0.25
	CP	0.59	0.5	0.43	0.65	0.77	0.5	0.85	0.64	1.69	0.68	0.65	0.74	0.56	0.58	0.47	0.44	0.25
	Days × CP	1.03	0.86	0.74	1.12	1.33	0.87	1.48	1.1	2.93	1.17	1.13	1.28	0.96	1.01	0.82	0.76	0.43
*P*-value	Days	<0.01	<0.01	<0.01	<0.01	<0.01	<0.01	<0.01	<0.01	0.03	<0.01	0.04	<0.01	<0.01	<0.01	<0.01	<0.01	<0.01
	CP	<0.01	<0.01	<0.01	<0.01	<0.01	<0.01	<0.01	<0.01	0.03	<0.01	<0.01	<0.01	<0.01	<0.01	<0.01	<0.01	<0.01
	Days × CP	<0.01	0.04	<0.01	<0.01	<0.01	<0.01	<0.01	<0.01	<0.01	<0.01	<0.01	<0.01	<0.01	<0.01	<0.01	0.01	<0.01

### Fecal Microbial AA Excretion

As shown in Table 6, the excretion of AA of fecal microorganisms of piglets on day 25 was lower than that on day 10 and day 45 (*p* < 0.01), and the excretion of TAA and NEAA of fecal microorganisms of piglets on day 25 was higher than that on days 10 and 45 (*p* < 0.0l). There was no significant difference in fecal microbial EAA excretion with the increase of feeding time (*p* > 0.05). The AA excretion of fecal microorganisms of piglets in the L-CP group was lower than that in the M-CP group and H-CP group (*p* < 0.01). The excretion of fecal microorganisms TAA, EAA, and NEAA increased with the increase of CP levels on day 10 (*p* < 0.01), and the excretion of TAA, EAA, and NEAA of the M-CP group were significantly higher than that of L-CP and H-CP on the day 25 (*p* < 0.01). Generally speaking, the emission of EAA was greater than that of NEAA. The interaction effects between feeding time and dietary protein level (Days × CP) were as follows: Asp (*p* < 0.01), Ser (*p* < 0.01), Glu (*p* < 0.01), Gly (*p* < 0.01), Ala (*p* < 0.01), Pro (*p* < 0.01), Thr (*p* < 0.01), Cys (*p* < 0.01), Val (*p* < 0.01), Met (*p* < 0.01), Ile (*p* < 0.01), Leu (*p* < 0.01), Tyr (*p* < 0.01), Phe (*p* < 0.01), Lys (*p* < 0.01), His (*p* < 0.01), and Arg (*p* < 0.01) in piglet feces.

**Table 6:** Effects of dietary protein levels on the microbe AA excretion in feces of piglets (mg/g DM).

**Time (d)**	**Items**	**Asp**	**Ser**	**Glu**	**Gly**	**Ala**	**Pro**	**Tyr**	**Thr**	**Cys**	**Val**	**Met**	**Ile**	**Leu**	**Phe**	**Lys**	**His**	**Arg**	**TAA**	**NEAA**	**EAA**	**EAA/NEAA**
10 d	L-CP	0.315	0.145	0.435	0.216	0.376	0.173	0.165	0.156	0.025	0.320	0.058	0.260	0.312	0.255	0.205	0.108	0.168	3.70	1.66	2.03	1.22
	M-CP	0.617	0.253	0.710	0.535	0.265	0.362	0.302	0.365	0.063	0.706	0.065	0.563	0.795	0.456	0.428	0.213	0.336	7.03	2.74	4.29	1.38
	H-CP	0.765	0.438	1.340	0.712	1.406	0.516	0.565	0.690	0.052	0.985	0.310	0.728	1.011	0.683	0.626	0.265	0.522	11.61	5.19	6.43	1.32
25 d	L-CP	0.213	0.118	0.265	0.150	0.263	0.087	0.083	0.223	0.010	0.172	0.018	0.153	0.240	0.136	0.136	0.070	0.085	2.44	1.10	1.34	1.29
	M-CP	0.680	0.155	0.658	0.346	0.670	0.168	0.150	0.268	0.020	0.297	0.097	0.335	0.498	0.266	0.280	0.135	0.232	5.25	2.67	2.58	1.25
	H-CP	0.352	0.132	0.352	0.216	0.330	0.108	0.125	0.230	0.035	0.198	0.048	0.241	0.295	0.17	0.155	0.090	0.12	3.20	1.48	1.71	1.23
45 d	L-CP	0.698	0.343	0.792	0.633	0.628	0.176	0.395	0.462	0.052	0.348	0.160	0.677	0.395	0.593	0.467	0.270	0.346	7.35	3.27	5.08	1.25
	M-CP	1.045	0.355	1.233	0.702	1.168	0.362	0.408	0.533	0.105	0.758	0.170	0.702	1.108	0.628	0.505	0.253	0.395	10.04	4.86	5.57	1.15
	H-CP	0.853	0.351	1.212	0.532	1.030	0.308	0.350	0.470	0.077	0.593	0.153	0.545	1.070	0.553	0.405	0.187	0.312	9.00	4.29	4.72	1.10
SEM	Days	0.011	0.012	0.016	0.009	0.018	0.008	0.014	0.012	0.004	0.013	0.005	0.090	0.014	0.009	0.008	0.006	0.01	0.044	0.057	0.011	
	CP	0.011	0.012	0.016	0.009	0.018	0.008	0.014	0.012	0.004	0.013	0.005	0.090	0.014	0.009	0.008	0.006	0.01	0.044	0.057	0.011	
	Days × CP	0.020	0.020	0.027	0.016	0.032	0.014	0.024	0.021	0.007	0.022	0.009	0.016	0.024	0.016	0.015	0.011	0.017	0.161	0.075	0.098	
*P*-value	Days	<0.01	<0.01	<0.01	<0.01	<0.01	<0.01	<0.01	<0.01	<0.01	<0.01	<0.01	<0.01	<0.01	<0.01	<0.01	<0.01	<0.01	<0.01	<0.01	0.04	<0.01
	CP	<0.01	<0.01	<0.01	<0.01	<0.01	<0.01	<0.01	<0.01	<0.01	<0.01	<0.01	<0.01	<0.01	<0.01	<0.01	<0.01	<0.01	<0.01	<0.01	<0.01	<0.01
	Days × CP	<0.01	<0.01	<0.01	<0.01	<0.01	<0.01	<0.01	<0.01	0.010	<0.01	<0.01	<0.01	<0.01	<0.01	<0.01	<0.01	<0.01	<0.01	<0.01	<0.01	<0.01

The excretion of amino acids except for Cys and Ile of fecal microorganisms increased with the increase of dietary protein level on day 10 (*p* < 0.01). The excretion of amino acids except for Cys and His of feces microbial in the M-CP group was higher than that in L-CP and H-CP (*p* < 0.01), and the excretion of microbial AA in L-CP was the lowest on day 25. The fecal excretion of amino acids except for Ser, Glu, Cys, Met, Ile, Leu, Tyr, and His of fecal microorganisms of piglets in the M-CP group was higher than that in the L-CP group and H-CP group (*p* < 0.01).

### Effects of Different Protein Levels on Microbial AA Composition in Feces and Ileum of Piglets

As shown in Tables 7, 8, the number of microbial AA increased with the increase of piglet age. The EAA/NEEA of Ile chyme and feces decreased with the increase of dietary CP (*p* < 0.01). The protein nutrition patterns of Asp, Ala, Glu, and Leu in the AA composition of ileum and feces of piglets were more obvious. The proportion of His, Tyr, Met, Cys, and Ser was lower than the average level, while the proportion of Phe, Leu, Pro, Ala, Glu, and Asp were higher than the average level. The interaction effects between feeding time and dietary protein level (Days × CP) were was reflected in Asp (*p* < 0.01), Glu (*p* < 0.01), Ala (*p* < 0.01), Pro (*p* < 0.01), Cys (*p* < 0.01), Val (*p* < 0.01), Met (*p* < 0.01), Ile (*p* < 0.01), Tyr (*p* < 0.01), Phe (*p* < 0.01), Lys (*p* < 0.01), His (*p* < 0.01), and Arg (*p* < 0.01) in ileum and feces of piglets.

**Table 7:** Effects of dietary protein levels on the microbial bacteria AA form in feces of piglets (%).

**Time (d)**	**Items**	**Asp**	**Ser**	**Glu**	**Gly**	**Ala**	**Pro**	**Tyr**	**Thr**	**Cys**	**Val**	**Met**	**Ile**	**Leu**	**Phe**	**Lys**	**His**	**Arg**	**TAA**	**NEAA**	**EAA**	**NEAA/EAA**
10 d	L-CP	8.56	3.93	11.76	5.87	10.25	4.62	4.36	4.27	0.67	8.67	1.55	7.050	8.52	6.92	5.57	2.90	4.54	100.00	45.0	55.0	1.22
	M-CP	8.33	3.43	9.62	7.23	8.49	4.87	4.06	4.92	0.84	10.05	0.88	7.630	10.73	6.18	5.80	2.91	4.55	100.00	42.0	58.0	1.38
	H-CP	6.60	3.80	11.54	6.15	12.13	4.45	4.88	5.94	0.44	8.31	2.69	6.300	8.72	5.89	5.41	2.30	4.47	100.00	44.7	55.3	1.32
25 d	L-CP	8.82	4.87	10.92	6.17	10.79	3.54	3.38	9.25	0.52	7.13	0.79	6.240	9.90	5.61	5.65	2.86	3.57	100.00	45.1	54.9	1.29
	M-CP	8.93	3.41	10.90	7.56	9.19	3.67	3.16	5.89	0.44	6.44	2.05	7.330	10.98	5.90	6.12	2.93	5.09	100.00	43.7	56.3	1.25
	H-CP	11.04	4.13	11.04	6.80	10.20	3.3	3.84	7.31	1.13	6.26	1.44	7.610	9.15	5.36	4.89	2.89	3.62	100.00	46.5	53.5	1.23
45 d	L-CP	9.54	4.64	10.79	8.62	8.53	2.4	5.38	6.27	0.72	4.73	0.99	9.180	5.33	8.09	6.38	3.69	4.72	100.00	44.5	55.5	1.25
	M-CP	10.01	3.41	11.80	6.72	11.20	3.46	3.91	5.13	1.01	7.26	1.67	6.710	10.62	6.05	4.83	2.42	3.80	100.00	46.6	53.4	1.15
	H-CP	9.49	3.91	13.47	5.89	11.46	3.42	3.88	5.20	0.85	6.58	1.70	6.070	11.92	6.15	4.51	2.06	3.45	100.00	47.6	52.4	1.10
SEM	Days	0.19	0.14	0.16	0.13	0.23	0.11	0.17	0.16	0.05	0.17	0.07	0.11	0.24	0.12	0.15	0.10	0.12		0.28	0.28	
	CP	0.19	0.14	0.16	0.13	0.23	0.11	0.17	0.16	0.05	0.17	0.07	0.11	0.24	0.12	0.15	0.10	0.12		0.28	0.28	
	Days × CP	0.32	0.24	0.27	0.23	0.40	0.19	0.30	0.28	0.09	0.29	0.11	0.19	0.41	0.20	0.20	0.17	0.21		0.49	0.49	
*P*-value	Days	<0.01	0.12	<0.01	<0.01	0.59	<0.01	<0.01	<0.01	0.02	<0.01	<0.01	0.09	0.06	<0.01	0.20	0.32	<0.01		<0.01	<0.01	<0.01
	CP	0.9	<0.01	<0.01	<0.01	<0.01	0.01	0.02	<0.01	0.08	<0.01	<0.01	<0.01	<0.01	<0.01	<0.01	<0.01	<0.01		<0.01	<0.01	<0.01
	Days × CP	<0.01	0.36	<0.01	<0.01	<0.01	<0.01	<0.01	<0.01	<0.01	<0.01	<0.01	<0.01	<0.01	<0.01	<0.01	<0.01	<0.01		<0.01	<0.01	<0.01

**Table 8:** Effects of dietary protein levels on the microbial bacteria AA form in ileum of piglets (%).

**Time (d)**	**Items**	**Asp**	**Ser**	**Glu**	**Gly**	**Ala**	**Pro**	**Tyr**	**Thr**	**Cys**	**Val**	**Met**	**Ile**	**Leu**	**Phe**	**Lys**	**His**	**Arg**	**TAA**	**NEAA**	**EAA**	**EAA/NEAA**
10 d	L-CP	9.41	3.64	11.61	5.46	7.23	3.93	4.52	5.26	0.46	7.44	0.41	7.19	13.41	7.17	4.78	3.32	4.78	100.0	42.3	58.7	1.39
	M-CP	9.89	3.39	11.47	7.05	6.42	3.88	4.11	5.64	0.46	6.70	0.28	6.69	12.59	7.33	5.47	3.46	5.19	100.0	42.1	57.9	1.38
	H-CP	8.76	3.60	12.05	8.05	7.25	4.98	3.47	4.92	0.44	8.68	0.40	6.81	12.19	6.46	4.75	2.99	4.22	100.00	44.7	55.3	1.24
25 d	L-CP	9.44	4.65	10.23	5.52	5.96	5.48	4.09	4.87	0.48	6.47	0.68	6.41	13.71	8.14	3.86	4.67	5.35	100.00	41.3	58.7	1.42
	M-CP	11.27	3.95	12.80	6.39	6.35	4.75	3.60	4.72	0.26	7.87	0.38	8.00	12.65	7.16	3.67	2.59	3.63	100.00	45.5	54.5	1.20
	H-CP	10.72	4.76	12.58	7.23	6.06	7.26	2.91	4.93	0.27	7.40	0.22	6.73	12.31	7.46	4.02	2.77	2.38	100.00	48.6	51.4	1.06
45 d	L-CP	10.92	4.25	9.77	5.82	6.53	4.93	2.96	5.02	0.48	7.34	0.63	8.32	12.03	8.51	4.10	4.62	3.74	100.00	42.2	57.8	1.37
	M-CP	10.37	5.43	11.96	6.43	7.07	4.57	3.04	4.67	0.55	6.28	0.35	6.27	12.60	6.46	4.82	4.37	3.14	100.00	47.5	52.5	1.11
	H-CP	10.80	6.15	12.58	7.53	7.80	4.44	3.87	5.37	0.42	6.47	0.47	7.08	10.09	6.34	3.86	2.77	3.13	100.00	49.3	50.7	1.03
SEM	Days	0.15	0.11	0.22	0.16	0.14	0.12	0.12	0.12	0.02	0.21	0.01	0.12	0.18	0.13	0.09	0.09	0.08		0.30	0.30	0.02
	CP	0.15	0.11	0.22	0.16	0.14	0.12	0.12	0.12	0.02	0.21	0.01	0.12	0.18	0.13	0.09	0.09	0.08		0.30	0.30	0.02
	Days × CP	0.26	0.19	0.38	0.28	0.28	0.24	0.20	0.21	0.04	0.36	0.02	0.21	0.31	0.23	0.16	0.16	0.14		0.52	0.52	0.03
*P*-value	Days	<0.01	<0.01	0.387	0.12	<0.01	<0.01	<0.01	0.04	<0.01	0.01	<0.01	0.15	0.03	0.05	<0.01	<0.01	<0.01		<0.01	<0.01	<0.01
	CP	<0.01	<0.01	<0.01	<0.01	0.04	<0.01	0.03	0.93	0.01	0.14	<0.01	0.04	<0.01	<0.01	<0.01	<0.01	<0.01		<0.01	<0.01	<0.01
	Days × CP	<0.01	<0.01	<0.01	0.35	<0.01	<0.01	<0.01	0.03	<0.01	<0.01	<0.01	<0.01	0.06	<0.01	<0.01	<0.01	<0.01		<0.01	<0.01	<0.01

## Discussion

### Apparent Digestibility of CP and AA

Research showed that the composition of dietary nutrients could directly affect the secretion of digestive enzymes in piglets (11). Griffiths reported that high protein levels could significantly affect the activity of digestive enzymes in the intestines of piglets (13). The secretion of digestive enzymes directly restricted the absorption and utilization of dietary CP, and so different dietary CP levels could lead to differences in fecal CP, AA, fecal CP digestibility, and AA digestibility of piglets. In this experiment, the apparent digestibility of CP and AA were increased linearly with the increase of dietary CP levels. When the CP levels increased to 20%, the measured indexes and the corresponding apparent digestibility increased significantly. It could be seen that increasing the dietary protein level in an appropriate range would directly affect the digestion and metabolism of nutrients such as dry matter, CP and AA in piglets.

In this study, the digestibility of dry matter, CP, and N increased significantly with the increase of feeding time, and H-CP was significantly higher than that of the L-CP group and M-CP group. The reasons were the following points: first, the intestinal development of piglets was not perfect, the secretion of digestive enzymes is less, the digestive system of piglets tended to mature after 45 days, and the secretion of all kinds of digestive enzymes tended to be stable. Secondly, properly increasing the dietary protein level and balanced AA level was beneficial for the absorption and utilization of the digestive tract of piglets. Some studies had shown that endogenous N could affect the apparent digestibility of intestinal N. When the dietary protein content was low, because of the increasing proportion of endogenous protein, the apparent digestibility of CP was decreased. In addition, protease was a kind of dietary protein metabolic enzyme, and the dietary protein level could directly affect the protease activity in the digestive tract. Within a certain range of protein levels, the apparent digestibility of CP was positively correlated with dietary protein levels (14), which was consistent with the results of this study.

There was not a simple linear relationship between CP apparent digestibility and dietary CP content. The study of Silva and Perera (15) pointed out that with the increase of dietary protein level, the apparent digestibility of dietary protein increased at first and then decreased. The results showed that there is an optimal concentration range for protein utilization. The digestibility of protein would not be ideal if the protein level was below or above the concentration range, that is, the protein digestibility would not be ideal on day 45. There was no such change in the results of this study, and the reason for the difference might be that the protein level in the study did not exceed the optimal range and belonged to the rising zone. At the same time, weaned piglets were in a special physiological period, and the utilization of protein level at this stage remains to be further studied.

The differences in dietary N digestion and utilization of piglets under different CP levels led to differences in N excretion (16, 17). In this study, within the range of 14–20% CP the apparent N digestibility of piglets increased linearly with the increase of CP levels, and the total N excretion increased with the increase of dietary CP levels and feeding time, which was consistent with the conclusions of other scholars (18). The apparent biological value of protein was mainly affected by the deposition ability of N nutrients and the degree of dietary protein balance, which reflected the utilization degree of N nutrients in piglets. The protein balance of the three diets used in this experiment was the same, and so the apparent biological value mainly depended on the ability of piglets to deposit N nutrients absorbed from the diet. When the dietary protein level was lower than the needs of the body, the piglets would make adaptive adjustments in order to improve the utilization efficiency of N nutrients absorbed in the diet and increase the deposition of N nutrients.

### Effects of Different Protein Levels on Microbial AA Composition in Feces and Ileum of Piglets

AAs played a very important role in animal nutrition and physiology (19). However, there was a lack of knowledge about the utilization and metabolism of AA in intestinal microorganisms. The results of human-like microorganisms *in vitro* culture showed that the rapidly fermented AA of human-like microorganisms was Lys, Arg, Thr, His, Glu, and Asp (20). The addition of fermentable carbohydrates to the culture system could reduce the decarboxylation of AA and the formation of amines by bacteria (21). The study also showed that the bacteria of Clostridium, Bacteroides, and Prepfococo were the main AA fermentation bacteria in the human intestine (22). Studies on fistula piglets had shown that Lys, Phe, and branched-chain AA synthesized by bacteria in the intestinal cavity can be absorbed by the small intestine and large intestine (23). However, these findings did not explain the high level of net utilization of AA in the intestine (24). So far, the metabolic pathway of dietary AA, especially essential AA, in the intestinal cavity was not clear (25). The study also showed that the bacteria of Clostridium, Bacteroides, and Prepfococo were the main AA fermentation bacteria in the human intestine (26). Studies on fistula pigs had shown that lysine, phenylalanine, and branched chain AA synthesized by bacteria in the intestinal cavity could be absorbed by the small intestine and large intestine (27, 28). However, these findings did not explain the high level of net utilization of AA in the intestine (27). So far, the metabolic pathway of dietary AA, especially essential AA, in the intestinal cavity was not clear (29). In this experiment, the proportion of microbial AA in piglet feces was only 1–5%, and the response of intestinal microorganisms Asp, Ala, Glu, and Leu to the N nutrition model was more obvious. In the AA composition of intestinal microorganisms, it was found that there were similarities between ileum and rectum: the proportion of His, Tyr, Met, Cys, and Ser was lower than the average level. The proportion of Phe, Leu, Pro, Ala, Glu, and Asp was higher than the average level.

## Conclusions

To sum up, this study revealed some scientific problems, such as the changes of N, CP, and AA excretion and digestibility of feces and microorganisms of piglets at different feeding times, the changes of AA composition of intestinal microorganisms, the changes in AA composition of microorganisms, the mechanism of enzyme activity, and the mechanism of enzyme activity affecting the changes of biogenic amines. The purpose of this work was to provide a theoretical basis for the follow-up research on how gastrointestinal microorganisms and gastrointestinal digestion and metabolism change the mode of N supply.

## Data Availability Statement

The original contributions presented in the study are included in the article/supplementary material, further inquiries can be directed to the corresponding author/s.

## Ethics Statement

The animal study was reviewed and approved by Sciences Animal Ethics Committee of Chinese Academy of Sciences (Hunan, China; Ethic approval number: SYXK 2014-0002).

## Author Contributions

ZT and ZY designed the whole experiment and verified the validity of experiment and checked the results. HD and TH performed the experiment, including chemical analysis, and statistical analysis. ZY and TH worked on the manuscript. ZS, ZG, PH, and ZR participated in the experiment design and gave valuable advice. All of the authors have read and approved the final version of this manuscript.

## Funding

This study was funded in part by the National Natural Science Foundation of China (31902167 and 31772610), Fundamental Research Funds for the Central Universities (XDJK2020C019), Chongqing Natural Science Foundation (Basic Research and Frontier Exploration Special Project) General Project (cstc2019jcyj-msxmX0524 and cstc2021jcyj-msxmX0966), Research project on educational and teaching reform in Southwest University (2019JY017), National Program on Key Basic Research Project of China (2013CB127303), and the National Science Foundation for postdoctoral scientists of China (2018M640895).

## Conflict of Interest

The authors declare that the research was conducted in the absence of any commercial or financial relationships that could be construed as a potential conflict of interest.

## Publisher's Note

All claims expressed in this article are solely those of the authors and do not necessarily represent those of their affiliated organizations, or those of the publisher, the editors and the reviewers. Any product that may be evaluated in this article, or claim that may be made by its manufacturer, is not guaranteed or endorsed by the publisher.

## Abbreviations

YSE: Yucca Schidigera Extract
CU: Candida Utilis
CON: control
RA: relative abundance
GLU: glucose
BUN: blood urea nitrogen
T-SOD: total superoxide dismutase
CAT: catalase
T-CHO: total cholesterol
AST: aspartate aminotransferase
MDA: transforming growth factor-β
ALT: alanine transaminase
T-AOC: total antioxidant capacity
ADG: average daily gain
ADFI: average daily feed intake.

## References

[1] Kendall DC Gaines AM Kerr BJ Allee GL. True ileal digestible tryptophan to lysine ratios in ninety- to one hundred twenty-five-kilogram barrows. J Anim Sci. (2007) 85:3004–12. 10.2527/jas.2007-0013 17686897

[2] Chen H Yi X Zhang G Lu N Chu L Thacker PA. Studies on reducing nitrogen excretion: I. Net energy requirement of finishing pigs maximizing performance and carcass quality fed low crude protein diets supplemented with crystalline amino acids. J Anim Sci Biotechnol. (2011) 2:84–93. 10.3382/ps.0310700

[3] Kerr B. Dietary manipulation to reduce environmental impact. In: Proceedings of the 9th International Symposium on Digestive Physiology in Pigs (Banff, AB) (2003).

[4] Shriver JA Carter SD Sutton AL Richert BT Senne BW Pettey LA. Effects of adding fiber sources to reduced-CP, amino acids-supplemented diets on nitrogen excretion, growth performance, and carcass traits of finishing pigs. J. Anim Sci. (2003) 81:492–502. 10.1046/j.1439-0396.2003.00411.x 12643494

[5] Shi B Liu J Sun Z Li TJ Zhu WW Tang Z. The effects of different dietary crude protein level on faecal crude protein and amino acid flow and digestibility in growing pigs. J Appl Anim Res. (2016) 46:74–80. 10.1080/09712119.2016.126057033187139

[6] Yang Z He T Bumbie G Hu H Chen QJ Lu C. Effects of dietary crude protein levels on fecal crude protein, amino acids flow amount, fecal and ileal microbial amino acids composition and amino acid digestibility in growing pigs. Animals. (2020) 10:2092–105. 10.3390/ani10112092 33187139 PMC7696704

[7] Dai ZL Zhang J Wu G Zhu WY. Utilization of amino acids by bacteria from the pig small intestine. Amino Acids. (2010) 39:1201–15. 10.1007/s00726-010-0556-9 20300787

[8] Zhou J Wang Y Zeng X Zhang T Li P Yao B. Effect of antibiotic-free, low-protein diets with specific amino acids compositions on growth and intestinal flora in weaned pigs. Food Funct. (2020) 11:493–507. 10.1039/C9FO02724F 31833513

[9] Horwitz W. Official methods of analysis of the association of official analytical chemists. In: Association of Official Analytical Chemists. 17th ed. (Arlington, VA) (2000). p. 1059–60.

[10] Moughan PJ Hodgkinson SM. The enzyme hydrolysed protein method for the determination of endogenous ileal nitrogen and amino acids flows-a modification. Anim Feed Sci Technol. (2003) 108:207–14. 10.1016/S0377-8401(03)00130-5

[11] Zhao Y Tian G Chen D Zheng P Yu B. Dietary protein levels and amino acid supplementation patterns alter the composition and functions of colonic microbiota in pigs. Anim Nutr. (2020) 6:143–51. 10.1016/j.aninu.2020.02.005 32542194 PMC7283365

[12] National Research Council. Washington, DC: National Research Council (2012).

[13] Griffiths D W Moseley G. The effect of diets containing field beans of high or low polyphenolic content on the activity of digestive enzymes in the intestines of rats. J Sci Food Agric. (2010) 31:255–9. 10.1002/jsfa.2740310307 6966014

[14] Nieto R Miranda A García M Aguilera JF. The effect of dietary protein content and feeding level on the rate of protein deposition and energy utilization in growing Iberian pigs from 15 to 50kg body weight. British J Nutr. (2002) 88:39–49. 10.1079/BJN2002591 12117426

[15] Silva SSD Perera M. Effect of dietary protein level and salinity with further observations on variability in daily digestibility. Agriculture. (1984) 38:293–306. 10.1016/0044-8486(84)90334-X

[16] Li TQ Hao YJ An RY Gao YJ. Effect of dietary protein level on digestibility of Amur Sturgeon larvae. Freshw Fish. (2002) 5:51–4. (In Chinese). 10.3969/j.issn.1000-6907.2002.05.020

[17] Zhang B Wang C Liu H Liu J Liu H. Effects of dietary protein level on growth performance and nitrogen excretion of dairy heifers. Asian Austral J Anim Sci. (2016) 30:386–91. 10.5713/ajas.16.0214 27554361 PMC5337918

[18] Li Y Wei H Li F Guo Q Duan Y Yin Y. Effects of low-protein diets supplemented with branched-chain amino acids on lipid metabolism in white adipose tissue of piglets. J Agric Food Chem. (2017) 65:2839–48. 10.1021/acs.jafc.7b00488 28296401

[19] Duan J Jie Y Ren W Liu T Cui Z Huang X. Dietary supplementation with L-glutamate and L-aspartate alleviates oxidative stress in weaned piglets challenged with hydrogen peroxide. Amino Acids. (2016) 48:53–64. 10.1007/s00726-015-2065-3 26255283

[20] Yin J Li T He J. Long-term effect of lysine restriction on liver global proteins, meat quality, and blood biochemical parameters in pigs. Protein Peptide Lett. (2018) 25:405–16. 10.2174/0929866525666180406142451 29623821

[21] Li Y Yin J Han H Liu G Deng D Kim SW. Metabolic and proteomic responses to long-term protein restriction in a pig model. J Agr Food Chem. (2018) 66:12571–9. 10.1021/acs.jafc.8b05305 30380847

[22] Zheng P Song Y Tian Y Zhang H Yu B He J. Dietary arginine supplementation affects intestinal function by enhancing antioxidant capacity of a nitric oxide-independent pathway in low-birth-weight piglets. J Nutr. (2018) 148:1751–9. 10.1093/jn/nxy198 30383283 PMC6209807

[23] Yin J Ying Y Han H Liu ZJ Zeng XF Li T. Long-term effects of lysine concentration on growth performance, intestinal microbiome, and metabolic profiles in a pig model. Food Funct. (2018) 9:4153–63. 10.1039/C8FO00973B 30058657

[24] Scott KP Gratz SW Sheridan PO Flint HJ Duncan SH. The influence of diet on the gut microbiota. Pharmacol Res. (2013) 69:52–60. 10.1016/j.phrs.2012.10.020 23147033

[25] David LA Maurice CF Carmody RN Gootenberg DB Button JE Wolfe BE. Diet rapidly and reproducibly alters the human gut microbiome. Nature. (2014) 505:559–63. 10.1038/nature12820 24336217 PMC3957428

[26] Metges C. Contribution of microbial amino acids to amino acids homeostasis of the host. J Nutr. (2000) 130:1857–64S. 10.1093/jn/130.7.1857S 10867063

[27] Torrallardona D Harris CI Fuller MF. Pigs' gastrointestinal microflora provide them with essential amino acids. J Nutr. (2003) 133:1127–31. 10.1093/jn/133.4.1127 12672930

[28] Torrallardona D Harris CI Fuller MF. Lysine synthesized by the gastrointestinal microflora of pigs is absorbed, mostly in the small intestine. AJP Endocrinol Metab. (2003) 284:E1177–80. 10.1152/ajpendo.00465.2002 12569087

[29] Wu G. Amino acids: metabolism, functions, and nutrition. Amino Acids. (2009) 37:1–17. 10.1007/s00726-009-0269-0 19301095

